# Original Communication

**Published:** 1869-06-01

**Authors:** 


### ORIGINAL COMMUNICATION.

LEAVENWORTH, April 10, 1869

DR. J. ADAMS ALLEN:

DEAR SIR:—A person, calling himself Dr. M. F. Turner, is traveling through Kansas with a letter, purporting to have been written by yourself, recommending him to the good offices of medical men.

The letter looks like a forgery to me, and I am certain that the person, though a cripple, is unworthy of the aid he is asking. If you will inform me, if the letter is not genuine, I will put the medical men of this State upon their guard against the man. Very truly yours,

C. A. LOGAN.

### CAVE CANEM.

The Editor's reply to Dr. Logan's letter with regard to one M. F. Turner, a traveling mendicant, was unfortunately omitted in making up the forms of the last number. Briefly, there is no greater nuisances than letters from physicians to others of the craft, endorsing such claims for charity. We have been personally bled *ad deliquium animi* in this way, and will never be a party to such an imposition on others. It is perhaps unnecessary to say that the letter referred to by Dr. Logan is a sheer forgery.

But we confess to have a dim recollection of having been bitten by a scamp who is well described by our friend Logan, who had his pockets stuffed with letters to the present writer.

